# Femtosecond laser-assisted implantation of corneal stroma lenticule for keratoconus

**DOI:** 10.1007/s10792-021-01739-8

**Published:** 2021-02-24

**Authors:** Adriano Fasolo, Alice Galzignato, Emilio Pedrotti, Chiara Chierego, Tiziano Cozzini, Erika Bonacci, Giorgio Marchini

**Affiliations:** 1 Department of Neurosciences, Biomedicine and Movement Sciences, Eye Clinic, U.O.C. di Oculistica–Policlinico G.B. Rossi, University of Verona, P.le L.A. Scuro 10, 37134 Verona, Italy; 2 The Veneto Eye Bank Foundation, Venezia, Italy

**Keywords:** Keratoconus, Additive keratoplasty, Femtosecond laser, Stromal keratophakia, Intrastromal inlay, Corneal stroma lenticule implantation

## Abstract

**Purpose:**

To review recent progress, challenges, and future perspectives of stromal keratophakia for the treatment of advanced keratoconus.

**Methods:**

We systematically reviewed the literature in the PubMed database, last update June 30, 2020. No language restriction was applied. The authors checked the reference lists of the retrieved articles to identify any additional study of interest.

**Results:**

Several techniques have been proposed for the treatment of keratoconus in order to avoid or delay keratoplasty. This was primarily due to the lack of accessibility to donor corneas in many countries. The ease and predictability of the more advanced femtosecond lasers used to correct ametropias by stromal lenticule extraction lead to hypothesize that generated refractive lenticules could be implanted into corneal stromal layers to restore volume and alter the refractive properties of the cornea in patients with corneal ectasias. At the same time, new techniques for preservation, customization, and cellular therapy of the corneal stromal have been developed, directing to the valorization of otherwise discarded byproducts such as donor corneas unsuitable for either lamellar of penetrating keratoplasty.

**Conclusions:**

Femtosecond laser-assisted stromal keratophakia could be a suitable therapeutic option for the treatment of corneal ectasias, especially in patients with advanced keratoconus, providing biomechanical support recovering the pachimetry to nearly normal value at the same time. The accuracy and predictability of the refractive outcome are yet a critical issue and the patient eligible for the procedure still has to be characterized.

## Introduction

Keratoconus (KC) is a bilateral, asymmetrical progressive ectatic disease of the cornea, resulting in corneal thinning, conical bulging, irregular astigmatism, and eventual Bowman’s layer breaks, hydrops, and scarring [1].

In 1965 Jose Ignacio Barraquer inaugurated the era of refractive keratoplasty, introducing the cornea remodeling by tissue addition and the concept of keratophakia describing the procedure of keratomileusis [from the ancient Greek κέρας (kéras: horn) and σμίλευσις (smileusis: carving)] by the use of intracorneal inlays [2, 3].

Based on Barraquer’s new perception, intrastromal additive techniques using synthetic inlays, such as ring segments, has been investigated in animal models and patients, as an option to alter the corneal refraction and as alternative to keratoplasty in ectatic diseases [4–6].

The introduction of femtosecond laser (FSL) in 2002 devised a new approach in refractive eye surgery, with improvement of safety, efficiency, and precision in several corneal surgical areas, including LASIK flap creation, intracorneal ring segment implantation, astigmatic keratotomy, presbyopia treatments, and intrastromal lenticule procedures [7, 8].

Very soon after that, benefitting from FSL advantages arose the concept of tissue addition within corneal stroma. In 2003, Jonas assessed the feasibility of the FSL-assisted intrastromal lamellar keratoplasty in ex vivo pig eyes, preserving intact Bowman’s layer and corneal epithelium [9]. Although lenticule creation was not possible with all FSL back in 2007, results of this study allow in vivo investigations and extensive optimization of FSL settings, aiming to the human in vivo application of the technique.

Studies in rabbits [10–13] and primates [14–19], using both autologous and allogeneic corneal stroma lenticules, confirmed FSL-assisted endokeratophakia as feasible and safe technique to restore corneal architecture, with good wound healing response, good integration in the stroma, the absence of inflammation and corneal haze, and long-term survival.

At the same time, the ongoing improvement in FSL expertise enabled to shape clear lenticules and to create precise stromal corneal pockets beneath the corneal stroma [20–22].

The increasing popularity of FSL in refractive lenticule extraction alternative to excimer laser ablation in refractive surgery (femtosecond lenticule extraction, FLEx; small-incision lenticule extraction, SMILE; refractive lenticule extraction, ReLEx) substantiated the characterization of the FSL techniques for corneal stromal lenticule extraction and allogeneic implantation secondary to preservation [23–25].

In 2013, Pradhan and coworkers first reported the feasibility of the intrastromal lenticule implantation to correct hyperopia in a 23-year woman, implanting an allogeneic lenticule retrieved from a myopic SMILE, observing clear cornea with regular intraocular pressure and endothelial cell count after 12 months [26].

In this article, we focus on the knowledge of intrastromal implantation of human corneal lenticules. The aim is to discuss the feasibility and predictability of results of corneal structure remodeling in patients with advanced KC basing on the published literature.

## Methods

### Identification of records

We systematically searched the literature using PubMed database (National Center for Biotechnology Information; https://www.ncbi.nlm.nih.gov/pubmed, latest search June 30, 2020). The search strategies were “’femtosecond’ AND (‘intrastromal’ AND ‘implantation’ NOT ‘ring’)” *n* = 44; “’femtosecond’ AND (‘intrastromal’ AND ‘pocket’)” *n* = 33; “’femtosecond AND (‘intracorneal’ AND ‘keratoconus’)” *n* = 60; “’femtosecond’ AND (‘intrastromal’ AND ‘lenticul*’)” *n* = 58; “’femtosecond’ AND ‘additive keratoplasty’ *n* = 32”.

No date or language restrictions were applied for the search.

### Screening and reviewing

**Fig. 1:**
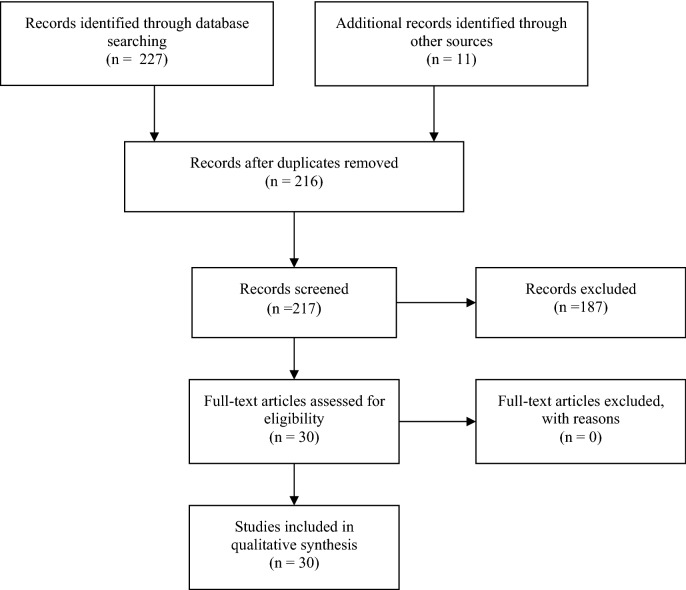
Study flow diagram

We excluded all duplicates and reviewed the abstracts of the remaining articles to ensure that they address the review question. The authors reviewed the full text of all the found publications and manually scanned the reference lists of selected retrieved articles to identify any additional study of interest. From the pool of the retrieved articles, we excluded those related to the application of FSL in corneal ring insertion, refractive inlays or epikeratophakia, keratoplasty or surgeries other than additive, and those reporting results in patients with eye disorders other than KC. Disagreements were resolved by discussion. Findings from the articles were summarize and included in the qualitative synthesis to described evidence of the state of the science related to the focus of the present study. The examined studies, methods, and results are summarized in Fig. 1.

## Results

### Intrastromal keratoplasty for keratoconus

#### Ex vivo studies on human corneas

Daamgard and coworkers used an ex vivo model of human donor corneas to examine the dependency of the lenticule thickness and implantation depth on the corneal curvature and the biomechanical strength at increased chamber pressure [27].

The study considered four groups of seven donor corneas each, with a combination of two implantation depths (110 and 160 μm) and two maximum thicknesses (95 and 150 μm) of 6.5 mm spherical biconcave lenticules collected by SMILE technique and directly implanted.

Axial keratometry showed that thicker lenticules tended to induce posterior flattening instead of anterior curvature steepening, with achieved correction generally lower than the power of the implanted lenticule. In addition, increased chamber pressure after implantation caused significant steepening of the anterior surface due to weakening of the corneal tissue, and consequently higher total corneal refractive power values in the 4-mm apex.

To increase corneal thickness and achieve central corneal flattening, an effect similar to that of intrastromal ring insertion, Mastropasqua, and Nubile implanted hyperopic-shaped lenticule, thinner in the central area and gradually thicker toward the peripheral part of the optic zone, into stromal pocket created in donor human corneas [28].

They compared two groups of six lenticules 30 µm thick in the center, with an optical zone of 5 mm and 6 mm and peripheral maximal thickness of 110 and 148 µm, respectively, implanted 115 µm under the Bowman’s layer. Group 1 showed an increase in corneal thickness at 3- and 6-mm ring from the corneal center of a mean (SD) of 120.33 (28.32) and 68.83 (38.23) µm, while Group 2 showed increases of 72.17 (21.77) and 125.17 (34.43) µm, respectively. A significant flattening of the central areas was obtained, with mean (SD) AvgK difference in the central 3 mm zone between pre- and post-implantation of − 7.31 (1.52) D, (*P* = 0.002), without differences between groups. The diameter of the flattened area correlated with the optical zone diameter of the implanted lenticule, with a flattening zone diameter of 3.03 (0.14) and 3.93 (0.51) mm in the group 1 and 2, respectively.

We assessed the feasibility of restoring corneal shape and thickness by a FSL-assisted small-incision intrastromal corneal lenticule addition focused on the restoration of a physiological corneal curvature of the posterior surface, choosing the depth of the dissection pockets close to the endothelial side [29]. First, we produced a model of ectatic human cornea to resemble ex vivo the topographic features of keratoconic corneas. By two subsequent excimer laser 97-μm ablations in the infero-temporal corneal quadrant, endothelial side, we thinned 17 human donor corneas mounted on an artificial anterior chamber and increased the chamber pressure to expose the ectasia. Then we shaped by FSL an equal number of biconvex stromal lenticules, with the same curvature radius on both surfaces, 200 µm thick and 6.5 mm wide, and implanted them into a stromal pocket obtained by FSL in the previously created ectatic corneas. We found mean (SD) central and thinnest corneal thickness of 887.41 (125.84) and 725.71 (91.60) µm, respectively (*P* < 0.0001), and flattening of posterior K1 and K2 of 5.42 (2.14) and 6.50 (2.75) D, respectively. Anterior and posterior astigmatism, anterior and posterior asphericity, and spherical aberration did not differ significantly compared to pre-intervention.

#### Clinical experience

Ganesh and coworkers studied the flattening effects, refraction and safety results of tissue addition by femtosecond intrastromal lenticular implantation (FILI) when combined with accelerated collagen crosslinking (CXL) in patients with progressive KC and contact lens intolerance [30].

With the aim to flatten the center of the cone, 6 cryopreserved SMILE-derived hyperopic lenticules 6.0–7.0 mm diameter and 120 µm thickness, were soaked in 0.25% riboflavin dye and punched 3 mm centrally resulting in a donut-shaped tissue. Lenticules were implanted into stromal pockets shaped by FSL (100 µm depth and 7.0–8.0 mm diameter) centered on the first Purkinje image, and exposed to UV-A radiation (30 mW/cm^2^) for 3.3 min.

Clinical improvements 6 months after surgery was obtained for uncorrected distance visual acuity [UDVA, from mean (SD) LogMAR of 1.06 (0.48)–0.38 (0.27)], corrected distance visual acuity [CDVA, from 0.51 (0.20) logMAR–0.20 (0.24)], and for manifest spherical equivalent (from − 3.47 (1.15) D to − 1.77 (1.7) D). A generalized flattening of mean keratometry and a decrease in the anterior Q value were achieved, with a reduction of high order aberrations due to more regular corneal surface. No intra- or postoperative complications were reported.

Although in a small number of eyes, this initial experience suggested that combining of CXL with a tissue addition surgical approach is feasible and may be a promising treatment option for clinical KC.

In 2017, Jadidi and Hasanpour reported the intrastromal corneal lenticule implantation in a 7-year-old girl who presented anterior protrusion and paracentral corneal thinning. Stromal donor lenticule (200 µm thick and 8.0 mm diameter, shape not reported) and recipient corneal pocket (150 µm depth and 9.5 mm diameter) were performed by FSL, and 1 month after surgery central Kmax decreased from 50.4 to 48.1 D, with clear cornea, and normal intraocular pressure and endothelial cell count [31].

Following the ex vivo study, Mastropasqua and Nubile investigated the effect of the FSL-assisted stromal lenticule addition keratoplasty (SLAK) in 10 patients with stage III and IV central KC [32]. Stromal lenticules with hyperopic profile obtained with FLEx procedure (6 mm optical zone and 0.7 mm transition zone; central and peripheral thickness of 30 and 148 µm, respectively) were implanted into FSL-shaped intrastromal pockets of recipient corneas. At day 7, the corneas were found clear and, compared to preoperative, at 6 months mean (SD) improvements were found for UDVA, from 1.58 (0.36) to 1.22 (0.37) logMAR and for CDVA, from 1.07 (0.17) to 0.70 (0.23) logMAR. Corneal topography at 6-month postoperative showed decreased Q values and a flattening of the cone with decreased anterior mean (SD) curvature from 58.69 (3.59) to 53.59 (3.50) D (AVG-K at 3 mm). AS-OCT pachimetry showed a significant increase in thickness of the central and mid-peripheral cornea produced by the lenticule implantation, with a mean (SD) CCT values increased from 406 (43) to 453 (39) μm at 6-month follow-up (*P* < 0.005).

Jadidi and coworkers reported the results obtained in four patients by the FSL-assisted intrastromal corneal graft (FAISCG), a new technique applied to obtain a corneal lenticule with precise diameter and thickness, and to shape intrastromal pockets as well [33].

Seven days after the procedure, all grafts were clear, and after 12 months no folds or interface complications were observed by Visante OCT, and UCVA improved in all patients.

Jin and coworkers compared retrospectively the efficacy of the small-incision FSL-assisted intracorneal concave lenticule implantation (SFII) with penetrating keratoplasty (PK) in 11 and 20 patients with progressive KC intolerant to contact lenses [34]. They obtained corneal graft lenticules of mean 250.41 μm thickness and 7.5 mm diameter from human donor corneas after FSL treatment and additional PRK shaping with a myopic correction of − 4.00 D. Then, intracorneal stromal pockets were shaped in the recipients by a SMILE myopic correction of − 0.75 D (28 μm thickness, 160 μm cap thickness, 7.8 mm cap diameter, and 7.8 mm corneal diameter) and during implantation the anterior–posterior orientation of the inlay was maintained.

At 24-month evaluation, total integration of the implant and the host stroma was observed in all recipients, with no visible tissue boundary. CDVA improved in all patients, with a majority of patients gaining 3 or more Snellen lines. In both groups, corneal topography showed a statistically significant decrease in the anterior K1 and K2.

Based on these results, Jin proposed SFII as a further option for the treatment of progressive KC by FSL-assisted stromal keratophakia. This technique not only increased recipient corneal thickness with good results in terms of changing corneal curvature and visual acuity, but also has a potential effects in the reduction of KC progression. As observed by Singh and coworkers, the addition of a lenticule into the corneal stroma would affect intraocular pressure estimation. It would be prudent to incorporate a correction factor while mentioning intraocular pressure in the small-incision femtosecond-laser-assisted intracorneal concave lenticule implantation group [35].

### Challenges and future directions: cryopreservation, customization, and cellular therapy of the corneal stroma

Perspective in secondary implantation of corneal lenticules extracted by FLEx and SMILE techniques for refractive purposes required adequate care in lenticules conservation in the short and long-term.

Storage condition, especially cryopreservation, demonstrated viability of isolated human keratocytes and maintenance of cellular structure after re-culture [36]. Test on human stroma ReLEx lenticules cryopreserved for 1 month, showed well alignment and preservation of the collagen structure, and good cellular viability, despite a decrease in collagen fibers density [23]. An initial clinical experience in eight hyperopic eyes and one aphakic eye of seven patients, who received 84–134 µm lenticules from ReLEx procedure with the standard technique implanted at 160 depth after cryopreservation, showed clear and stable lenticules within 48 h, and absence of shift up to the 6-month follow-up. [37] These results seem promising for a possible applications in patients with KC.

A further important field of research is the customization of lenticules, to modify the refractive power and widening the therapeutic options for additive intrastromal surgery. Damgaard and coworkers, in the effort to maximize the use of the byproducts of SMILE explored a way to modify lenticule with phototherapeutic keratectomy (PTK). They assessed the biological response of 7 SMILE-derived stromal lenticules treated with PTK compared with 7 non-lasered lenticules used as a control group, in terms of changes in cell density and morphology [38]. Despite some differences in lipid profile and collagen structure, they showed the feasibility of excimer laser in thinning and reshaping lenticules for volume restoration purposes in patients with hyperopia, keratoconus, presbyopia, aphakia and corneal dystrophies.

Finally, an attractive prospective is offered by cell therapy. To increase biocompatibility of stromal lenticules and their function for tissue repair and remodeling, decellularization showed promising results in the obtaining a cell-free scaffold and the elimination of genetic materials, while maintaining the extracellular matrix protein content and preserving mechanical and optical properties [39, 40]. Additionally, corneal stroma enhancement can be obtained by corneal stroma recellularization, based on the aptitude of mesenchymal stem cells (MSCs) to differentiate into adult keratocytes in vitro and in vivo [41]. Studies in animal model showed that synthetic and human corneal matrix seeded with human adipose-derived MSCs integrate functionally without induction of inflammatory reactions and new collagen production within the host stroma [42–45]. New collagen production was observed as patchy hyper reflective areas at the level of the stromal pocket after autologous adipose-derived adult stem cell (ADASC) implantation within the corneal stroma of patients with advanced keratoconus [46].

A study from Aliò et al. in patients with advance KC showed encouraging results. Five patients received anterior stromal allogeneic corneal laminas decellularized by the use of enzymes, 120 μm thick and 9 mm large, and four patients received corneal laminas recellularized with autologous adipose-derived adult stem cells by the injection of about 2 × 10^5^ cells in the graft followed by five days of cell culture. Three months after surgery, no clinical haze or scarring were observed, and at 6-month patients showed a general improvement of all visual parameters and all anterior keratometric values. Results after 12 months showed a mean improvement of corneal thickness of 116.4 μm in the central thickness at optical at coherence tomography scans [47, 48].

This study showed that the transplantation of decellularized corneal stroma obtained by the use of enzymes is safe (no complication recorded and no inflammatory response observed) and that adipose-derived MSCs survive in vivo into the human corneal stroma, with host keratocyte recellularization achieved and modest improvement in visual parameters and neo-collagen production. No differences were found between the two groups, so the future potentiality of stem cells deserves other studies.

## Discussion

Since the second half of the last century, ophthalmic surgeons investigated how to manage diseased corneas overcoming the well-established full thickness graft technique, aiming to obtain a less invasive intervention for the patient and the greatest utilization of the precious gift of donated corneas. In the surgical management of KC, anterior lamellar keratoplasty (ALK) revolutionized the transplant techniques [49] proving clinical results as good as penetrating graft, in terms of corneal clarity and refraction, while preserving recipient’s Descemet membrane and endothelial cells, reducing the risk of graft failure due to rejection or endothelial decompensation [50, 51].

The current florilegium of techniques to obtain anterior and posterior partial corneal grafts confirms they succeed [52–55].

However, despite the effort on public education and media toward corneal donation and the organization of eye banks, shortage of human donor tissues, which is a matter of concern in many countries, challenges ophthalmologists around the world [50, 55].

About 53% of the world’s population has no access to corneal transplantation, and approximately 12.7 million people were awaiting a transplantation at the time of the global survey conducted in the years 2012–2013 [56]. Xenotransplantation using fresh porcine cornea has been recently suggested as a feasible alternative to overcome the shortage of human donor corneas [57].

In the treatment of the KC, the second indication to transplantation worldwide (27%) [55], ophthalmic surgeons continue to keep up their effort to improve keratoplasty or find alternatives to the excision of the diseased cornea, with the purpose to assure safety and successful results to patients.

The application of CXL is an example, and in patients with progressive KC, it demonstrated to reduce effectively the need of corneal graft because of halting the development of the ectasia in the long-term [58].

FSL has been also introduced as a potentially valuable approach, although the disadvantage of suction and applanation of the cone during trephination, with intraoperative pitfalls and high postoperative astigmatism, are major limitations [59]*.*

Coullet J and coworkers obtained imprecise anatomic and refractive outcomes in lamellar graft, shaped by microkeratome and implanted beneath a nasal-hinged flap in the host cornea (microkeratome-assisted additive stromal keratoplasty, MASK) [60], while Carriazo and Cosentino obtained encouraging results by reshaping the ectatic cornea by means of crescent keratectomy performed with an excimer laser [61]. However, MASK and excimer laser ablation was found not successful substitutes of keratoplasty in the management of KC.

Conversely, the application of FSL in the stromal keratophakia to manage KC, focused in this review, could represent a realistic promising surgical approach for patients.

Current publications report early results on feasibility and safety, ensuring clear lenticules after surgery and in the long term, predictable corneal reshaping, negligible adverse reactions and, compared to keratoplasty, possible advantages in terms of reduced invasiveness, short surgery time, and use of topical anesthesia.

Reduced rejection rate is also expected because antigenic load to elicit an immunological response is lower, since only stromal keratocytes and collagen protected in stromal pockets are transplanted, without contact with tears, limbal vessels, or aqueous humor. However, stroma allograft rejection can occur, and studies on the decellularization of the lenticules aim to further reduce or withdraw immune response without affecting the collagen architecture [44].

The accuracy and predictability of the refractive outcome are yet a crucial issue and the proper patient for this procedure still has to be identified. Reinforce of the corneal architecture by recovering of the pachimetry to near-normal value by FSL-assisted stromal keratophakia could be rational in patients with KC, leading to a reduced risk of corneal perforation and making possible subsequent refractive surgeries and CXL, while changing in corneal curvature could improve contact lens fitting, thus avoiding the need of keratoplasty.

An open field of research that will deserve further studies is the depth of the stromal pocket, while lenticule thickness, shape, and diameter still have to be defined to maximize the efficacy of the corneal stroma remodeling in terms of central corneal profile, corneal aberration and final visual acuity.
